# Carry-over influence of maternal L-carnitine during gestation and lactation on metabolic response of New Zealand neonatal rabbits

**DOI:** 10.5194/aab-69-585-2026

**Published:** 2026-10-19

**Authors:** Shahzada Khurram Adrian Shah, Hamayun Khan, İsmail Ülger, Rasha Alonaizan, Shabana Naz, Suliman Alomar, Noha A. Tashkandi, Ala Abudabos, Luciana Rossi, Rifat Ullah Khan

**Affiliations:** 1 College of Veterinary Sciences, Faculty of Animal Husbandry & Veterinary Sciences, The University of Agriculture, Peshawar, Pakistan; 2 Department of Animal Science, Faculty of Agriculture, Erciyes University, Kayseri, Türkiye; 3 Department of Zoology, College of Sciences, King Saud University, Riyadh, 11451, Saudi Arabia; 4 Department of Zoology, Government College University, Faisalabad, Pakistan; 5 Doping Research Chair, Zoology Department, College of Science, King Saud University, Riyadh University, Riyadh, 11451, Saudi Arabia; 6 Institute of Research and Consulting, King Abdulaziz University, Jeddah, 21589, Saudi Arabia; 7 Department of Food and Animal Sciences, College of Agriculture, Tennessee State University, Nashville, TN 37209, USA; 8 Department of Veterinary Medicine and Animal Sciences (DIVAS), University of Milan, Milan, Italy

## Abstract

This study evaluated the effects of maternal L-carnitine supplementation on the growth performance, metabolic profile, and physiological responses of neonatal rabbits. A total of 160 5-month-old nulliparous pregnant New Zealand White does (2.84 ± 0.05 kg) were randomly assigned to four treatments: control (no supplementation) and L-carnitine-supplemented groups at 100, 200, and 300 mg L^−1^ in drinking water. Supplementation was provided throughout gestation and lactation to assess maternal carry-over effects on offspring. Neonatal growth performance was monitored through litter size, body weight, and average daily gain (ADG). Blood samples from neonates were collected during weeks 1, 2, and 3 for biochemical, hormonal, and oxidative stress analyses. Data were analysed using one-way ANOVA under a completely randomised design. Results showed that maternal L-carnitine supplementation improved litter performance and neonatal growth, with the highest body weight and ADG observed in the 300 mg L^−1^ group. Serum glucose levels were significantly elevated in the highest supplementation group across all weeks. Cholesterol and triglyceride concentrations were not significantly affected. Aspartate aminotransferase (AST) and alanine aminotransferase (ALT) were reduced in the LC-300 group during later weeks, suggesting improved hepatic status. Cortisol levels remained unchanged among treatments. However, triiodothyronine (T3) and thyroxine (T4) concentrations were significantly enhanced, particularly at higher supplementation levels, indicating improved metabolic activity. Malondialdehyde (MDA) levels were not significantly influenced, suggesting no adverse effect on oxidative status. In conclusion, maternal L-carnitine supplementation, particularly at 300 mg L^−1^, enhances the growth performance and metabolic efficiency of neonatal rabbits without negatively affecting physiological health.

## 1 Introduction

Rabbit production is an important component of livestock farming because of rabbits' high reproductive efficiency, rapid growth rate, and ability to provide high-quality animal protein (Abuelhamd et al., 2023; Al-Farraj et al., 2024; Goswami et al., 2025). The productivity and survival of rabbit neonates are strongly influenced by maternal nutrition during gestation and lactation as the nutritional status of the doe affects fetal development, milk production, and the physiological condition of offspring during the early stages of life (Symeon et al., 2015; Rizvi et al., 2025; Yu et al., 2025). Therefore, improving maternal nutrition through dietary supplementation has become an important strategy to enhance neonatal growth, health, and future performance (Xie et al., 2023; Puspani et al., 2025).

Despite increasing interest in nutritional programming, relatively few studies have investigated how maternal dietary interventions influence the metabolic and physiological development of rabbit offspring during the early postnatal period.

L-carnitine is a naturally occurring compound that plays a central role in energy metabolism by transporting long-chain fatty acids into the mitochondria for β -oxidation and energy production (Virmani et al., 2022; Merdana et al., 2025). In addition, L-carnitine has been associated with improved glucose utilisation, lipid metabolism, endocrine function, and antioxidant defence (Kraemer et al., 2008; Shahouzehi et al., 2022). Previous studies in different animal species have shown that dietary L-carnitine supplementation can improve reproductive performance, growth efficiency, immunity, and metabolic health (Zamani et al., 2023; Ulinski et al., 2023; Mpwaga et al., 2026). In breeding females, maternal supplementation may also support nutrient transfer to the developing fetus and newborn, thereby enhancing neonatal vitality and physiological adaptation after birth (González-Fernández et al., 2024). However, most previous investigations have focused on direct dietary supplementation of growing animals or reproductive performance of breeding females, whereas the potential carry-over effects of maternal L-carnitine supplementation on neonatal metabolic programming and physiological adaptation remain poorly understood in rabbits.

The neonatal period in rabbits is particularly sensitive as kits undergo rapid metabolic and physiological changes while relying heavily on maternal milk and body reserves (Habeeb et al., 2023). Assessment of blood biochemical and hormonal parameters such as glucose, cholesterol, triglycerides, liver enzymes (AST and ALT), cortisol, thyroid hormones (T3 and T4), and malondialdehyde (MDA) can provide valuable information regarding the energy metabolism, liver function, stress response, thyroid activity, and oxidative status of neonates (Pinto-Pinho et al., 2023). However, limited information is available regarding the effects of maternal L-carnitine supplementation on these physiological traits in rabbit offspring. Moreover, no previous study has comprehensively evaluated the dose-dependent effects of maternal L-carnitine supplementation on neonatal growth performance together with metabolic, hepatic, endocrine, and oxidative stress biomarkers during the first 3 weeks of postnatal life, a critical period for physiological and metabolic development.

Therefore, the present study was conducted to evaluate the influence of maternal supplementation of L-carnitine at levels of 100, 200, and 300 mg L^−1^ on the serum biochemical, hormonal, and oxidative stress parameters of rabbit neonates during the first 3 weeks of life. Unlike previous studies that primarily evaluated growth or reproductive responses, the present investigation was designed to simultaneously examine the maternal carry-over effects of graded L-carnitine supplementation on neonatal growth performance, glucose and lipid metabolism, liver function, thyroid activity, stress physiology, and oxidative status. The study specifically examined whether maternal L-carnitine supplementation could improve the metabolic efficiency, endocrine balance, and overall health status of rabbit neonates and aimed to identify the optimum supplementation level capable of maximising neonatal physiological development without adverse metabolic consequences.

## 2 Materials and methods

### 2.1 Animals, housing, and diets

A total of 160 5-month-old nulliparous pregnant New Zealand White does were used in this study. All maternal does were maintained under identical management conditions, including the same diet, husbandry practices, estrus synchronisation, artificial insemination, and parturition management. The average body weight of the does was 2.84 ± 0.05 kg. Animals were randomly allocated into four treatment groups, with each treatment consisting of 40 does. Within each treatment, does were further subdivided into five replicates, and each replicate consisted of eight does housed collectively in one cage unit. Therefore, a total of 20 replicate cage units (5 replicate cages × 4 treatments) were used in the experiment.

During gestation, each replicate group (eight does) was housed in a floor-mounted galvanised wire-mesh colony cage or park measuring 200 × 150 × 60 cm (length × width × height), providing approximately 0.375 m^2^ floor space per doe. The cages were installed in a naturally ventilated rabbit house under standardised environmental conditions. Plastic slatted flooring was used to facilitate hygiene and manure removal while minimising foot lesions. Each colony cage was equipped with a linear feeder and an independent nipple-drinker system to allow group access to feed and water ad libitum. The collective housing system was selected to reduce stress and permit normal social interaction among does before parturition. All replicate cage units were identical in design, stocking density, and environmental exposure to avoid housing-related bias among treatments. Throughout the gestation period, all does received the same basal pelleted diet formulated to meet the nutrient requirements of breeding rabbits, and feed was offered ad libitum. Fresh, clean drinking water was continuously available through the nipple-drinker system, except for the addition of L-carnitine in the respective treatment groups. Figure S1 in the Supplement shows the housing systems for rabbit does: collective gestation cages (eight pregnant does per replicate) and individual maternity cages for does with their litters after kindling, with cage dimensions, feeders, nipple drinkers, and plastic-slatted flooring.

The control group received unsupplemented drinking water, whereas group 1, group 2, and group 3 received L-carnitine (L-carnitine, ≥ 98 % purity, Guilin Co., Ltd., Guilin, Guangxi, China) supplementation in drinking water at concentrations of 100 (LC-100), 200 (LC-200), and 300 mg L^−1^ (LC-300), respectively. The supplementation levels were selected based on previous studies demonstrating beneficial physiological and productive responses to L-carnitine in rabbits and other livestock species while also allowing evaluation of a dose-dependent response within a biologically safe and practically applicable range. Each replicate cage was provided with a separate drinking system to ensure accurate administration of the assigned treatment concentration. Fresh L-carnitine-treated water was prepared daily and supplied ad libitum to the respective replicate groups throughout the experimental period. Water consumption was monitored regularly throughout the study. Supplementation commenced immediately after pregnancy confirmation (10–12 d after mating) and was continued throughout the remaining gestation period and the entire lactation period to evaluate the maternal carry-over effects on neonatal rabbits. Supplementation was provided during both gestation and lactation to evaluate the maternal carry-over effects on neonatal rabbits.

Natural mating was carried out by transferring receptive does individually to the cage of fertile bucks to avoid fighting and to ensure successful copulation. Pregnancy was confirmed by abdominal palpation approximately 10–12 d after mating. One week before the expected kindling date, pregnant does were removed from the collective colony cages and transferred to individual maternity cages to prevent aggression and to ensure accurate monitoring of litter performance and maternal traits.

After kindling, each doe, together with its litter, was housed individually in galvanised wire-mesh cages (60 × 50 × 40 cm) equipped with nipple drinkers and feeders under environmentally controlled conditions (20–25 °C temperature, 60 %–70 % relative humidity, and a 16 h light : 8 h dark photoperiod). Nest boxes were provided to ensure adequate nursing and protection of kits. No cross-fostering or litter equalisation (standardisation) was performed, and each litter remained with its biological mother throughout the suckling period to evaluate the true maternal carry-over effects of L-carnitine supplementation. Neonatal rabbits were allowed to suckle naturally without cross-fostering. Feed and fresh drinking water were offered ad libitum, and strict hygienic and biosecurity measures were maintained throughout the trial. The ingredients and chemical composition of the experimental diets are presented in Table 1.

**Table 1:** Composition and chemical analysis of experimental diets.

Item	%, as Fed
Alfalfa meal	32.00
Corn	30.00
Wheat bran	20.00
Rice bran	9.81
Soybean meal	6.00
Calcium bicarbonate	1.00
L-lysine	0.30
L-threonine	0.10
Tryptophan	0.01
Choline chloride	0.15
DL-methionine	0.08
Sodium chloride	0.20
Mineral-vitamin premix^*^	0.35
Chemical composition	Value
Digestible energy (MJ kg^−1^)	11.17
Dry matter, %	84.91
Crude protein, %	15.94
Ether extract, %	4.23
Ash, %	4.88
Ca, %	0.89
Total phosphorus, %	0.83

^*^ Mineral and vitamin premix supplied per kg of diet: Cu 3 mg, Zn 70 mg, Mn 2.5 mg, Fe 50 mg, Se 0.1 mg, I 0.5 mg, vitamin A 12 000 IU, vitamin D 900 IU, vitamin E 40 IU, vitamin K 30.5 mg, vitamin B6 0.6 mg, vitamin B12 0.003 mg, vitamin B1 0.2 mg, vitamin B2 1.6 mg, folic acid 0.05 mg, nicotinic acid 3.0 mg.

The litter size, individual body weight (BW), and average daily gain (ADG) of neonatal rabbits were recorded during the experimental period. Although body weight and growth-related parameters were measured repeatedly over time, statistical analysis was conducted using the average values obtained during the experimental period rather than a repeated-measures analytical model.

### 2.2 Sampling and analysis

Blood samples were collected from representative rabbit neonates randomly selected from each treatment group during the first, second, and third weeks of age [control litter size = 7.77; LC-100 = 7.97; LC-200 = 8.11; LC-300 = 7.94]. Two neonatal rabbits per replicate were randomly selected and humanely decapitated for blood collection at each sampling period. Blood samples were collected immediately after decapitation in sterile tubes without anticoagulant. The samples were allowed to clot at room temperature and then centrifuged at 3000 rpm for 15 min to separate serum. The harvested serum was stored at - 20 °C until biochemical analysis. Serum glucose (Bioscien ARENA Glucose Testing Kit), total cholesterol (AMP Diagnostics, Austria), and triglyceride (AMP Diagnostics, Austria) concentrations were determined using commercial diagnostic kits according to the manufacturers' instructions and analysed using a spectrophotometer (IRMECO Model U2020). Serum total protein was also determined using a commercial kit (AMP Diagnostics, Austria). Serum aspartate aminotransferase (AST) and alanine aminotransferase (ALT) activities were measured spectrophotometrically as indicators of liver function using commercial assay kits (AMP Diagnostics, Austria) with the IRMECO Model U2020 spectrophotometer according to the manufacturers' protocols. Serum cortisol, triiodothyronine (T3), and thyroxine (T4) concentrations were determined using commercial rabbit-specific enzyme-linked immunosorbent assay (ELISA) kits and analysed using an automatic ELISA microplate reader (Mindray MR-96A, Shenzhen Mindray Bio-Medical Electronics Co., Ltd., Shenzhen, China) according to the manufacturer's instructions. All serum samples and standards were analysed in duplicate, and the average value was used for statistical analysis. The intra-assay coefficient of variation (CV) was maintained below 10 % in accordance with the kit quality-control recommendations. Malondialdehyde (MDA) concentration was estimated as an indicator of lipid peroxidation and oxidative stress using thiobarbituric acid reactive substance (TBARS) methodology (Hafeez et al., 2024; Trung and Nhan, 2025). The absorbance of the TBARS reaction was measured spectrophotometrically using the IRMECO Model U2020, and MDA concentrations were calculated according to the standard procedure. All biochemical analyses were performed according to the manufacturers' protocols under identical laboratory conditions with appropriate quality-control samples included in each analytical batch.

### 2.3 Statistical analysis

The experiment was conducted using a completely randomised design (CRD) consisting of four maternal dietary treatment groups with five replicates per treatment. Each replicate consisted of eight does housed collectively, and the replicate cage ( n=5 per treatment) was considered to be the experimental unit for all maternal performance variables. For neonatal variables, the offspring originating from each replicate cage constituted the experimental unit, and measurements were analysed according to their respective maternal treatment groups. Neonatal measurements were obtained from offspring originating from the respective maternal treatment groups. Parameters recorded repeatedly over time, including neonatal body weight and average daily gain during the first, second, and third weeks of age, were analysed using a repeated-measure mixed-model procedure. In this model, maternal dietary L-carnitine treatment (0, 100, 200, and 300 mg L^−1^), week of measurement (weeks 1, 2, and 3), and the treatment × week interaction were included as fixed effects, whereas replicate nested within treatment was included as a random effect. Time (week) was treated as the repeated factor. An autoregressive covariance structure [AR(1)] was selected for the repeated-measure analysis based on the lowest Akaike Information Criterion (AIC) value as it provided the best fit for the covariance among repeated observations over time. When the ANOVA indicated a significant treatment effect ( P < 0.05), post hoc multiple comparisons among treatment means were performed using Duncan's Multiple Range Test. Data are presented as mean ± standard error of the mean (SEM), and statistical significance was declared at P < 0.05. All statistical analyses were performed using IBM SPSS Statistics software (Version 21.0; IBM Corp., Armonk, NY, USA).

## 3 Results

Maternal supplementation with L-carnitine positively influenced growth performance of dams and neonatal rabbits (Table 2). Litter size at 21 d was numerically improved in the supplemented groups compared with the control group, with the greatest response observed in the LC-200 group. Individual body weight at 21 d was higher in all L-carnitine-treated groups than in the control, while the LC-300 group showed the highest body weight. Likewise, average daily gain during 0–21 d increased in supplemented groups, with the greatest gain recorded in LC-300, followed closely by LC-100 and LC-200.

**Table 2:** Growth performance of dams and neonatal rabbits receiving maternal supplementation.

	Control	LC-100	LC-200	LC-300
Litter size at 21 d (n)	7.77 ± 0.14	7.97 ± 0.33	8.11 ± 0.23	7.94 ± 0.11
Individual body weight at 21 d (g)	3.45 ± 0.21	3.71 ± 0.18	3.67 ± 0.23	3.75 ± 0.32
Average daily gain during 0–21 d (g d^−1^)	47.62 ± 3.42	60.00 ± 2.56	58.10 ± 1.89	61.90 ± 1.99

LC-100, LC-200, and LC-300 were supplemented L-carnitine at rates of 100, 200, and 300 mg L^−1^, respectively.

The serum glucose concentration of rabbit neonates was significantly affected by maternal L-carnitine supplementation (Table 3). During the first week, the LC-300 group exhibited higher glucose concentration than the control and other lower-dose groups. In the second week, glucose concentration remained elevated in LC-300 compared with the control, whereas intermediate groups showed moderate responses. By the third week, LC-300 maintained the highest glucose level, while the remaining supplemented groups were comparable to the control.

**Table 3:** The effect of maternal L-carnitine supplementation on serum glucose (mg dl^−1^) concentration in rabbit neonates.

Groups	First week	Second week	Third week
Control	48.15 ± 2.89^b^	87.70 ± 2.26^b^	75.25 ± 0.78^b^
LC-100	57.54 ± 5.62^ab^	95.41 ± 1.43^ab^	74.13 ± 0.18^b^
LC-200	54.00 ± 3.12^b^	90.65 ± 1.10^ab^	79.77 ± 4.26^b^
LC-300	61.22 ± 4.61^a^	98.53 ± 0.13^a^	97.91 ± 1.56^a^

LC-100, LC-200, and LC-300 were supplemented L-carnitine at rates of 100, 200. and 300 mg L^−1^, respectively. Different superscripts in a column differ significantly ( P < 0.05).

**Table 4:** The effect of maternal L-carnitine supplementation on serum cholesterol (mg dl^−1^) concentration in rabbit neonates.

Groups	First week	Second week	Third week
Control	50.12 ± 2.06^ab^	53.95 ± 4.28^a^	53.68 ± 1.77^a^
LC-100	51.16 ± 1.04^ab^	43.18 ± 2.76^a^	51.20 ± 5.81^a^
LC-200	57.25 ± 4.02^a^	51.37 ± 1.25^a^	56.71 ± 1.37^a^
LC-300	49.14 ± 3.19^b^	48.12 ± 2.09^a^	50.31 ± 1.74^a^

LC-100, LC-200, and LC-300 were supplemented L-carnitine at rates of 100, 200, and 300 mg L^−1^, respectively. Different superscripts in a column differ significantly ( P < 0.05).

Serum cholesterol concentration showed limited changes among treatments (Table 4). In the first week, LC-200 showed higher cholesterol concentration, whereas LC-300 was lower than some other groups. However, no significant differences were observed among treatments during the second and third weeks.

Serum triglyceride concentration was not significantly influenced by maternal L-carnitine supplementation throughout the experimental period (Table 5). Although numerical variations were observed among groups, all treatments remained statistically comparable during the first, second, and third weeks.

**Table 5:** The effect of maternal L-carnitine supplementation on serum triglyceride (mg dl^−1^) concentration in rabbit neonates.

Groups	First week	Second week	Third week
Control	106.26 ± 7.10^a^	102.33 ± 10.76^a^	119.81 ± 1.11^a^
LC-100	104.37 ± 1.52^a^	115.63 ± 13.42^a^	113.81 ± 3.31^a^
LC-200	112.50 ± 1.59^a^	133.64 ± 28.60^a^	118.01 ± 0.78^a^
LC-300	114.61 ± 6.69^a^	119.43 ± 5.83^a^	122.78 ± 6.50^a^

LC-100, LC-200, and LC-300 were supplemented L-carnitine at rates of 100, 200, and 300 mg L, respectively. Different superscripts in a column differ significantly ( P < 0.05).

**Table 6:** The effect of maternal L-carnitine supplementation on serum aspartate aminotransferase or AST (U L^−1^) concentration in rabbit neonates.

Groups	First week	Second week	Third week
Control	18.11 ± 2.10^a^	21.47 ± 0.38^a^	18.12 ± 0.10^a^
LC-100	17.50 ± 0.73^a^	20.19 ± 0.86^ab^	19.52 ± 0.20^a^
LC-200	18.50 ± 1.05^a^	21.27 ± 0.60^a^	20.56 ± 1.11^a^
LC-300	18.03 ± 0.19^a^	18.72 ± 0.28^b^	18.46 ± o.57^b^

LC-100, LC-200, and LC-300 were supplemented L-carnitine at rates of 100, 200, and 300 mg L^−1^, respectively. Different superscripts in a column differ significantly ( P < 0.05).

Serum AST activity was affected during later sampling periods (Table 6). No differences were detected in the first week. In the second week, LC-300 showed lower AST activity compared with the control and some other groups. In the third week, LC-300 again exhibited reduced AST activity relative to the control and LC-200, indicating a possible protective effect on hepatic function.

Serum ALT activity was generally unaffected by maternal supplementation during the first and second weeks (Table 7). In the third week, however, LC-300 demonstrated lower ALT activity compared with the control group, whereas the remaining treatments were intermediate.

**Table 7:** The effect of maternal L-carnitine supplementation on serum alanine aminotransferase or ALT (U L^−1^) concentration in rabbit neonates.

Groups	First week	Second week	Third week
Control	21.56 ± 0.23^a^	21.70 ± 1.59^a^	28.56 ± 0.11^a^
LC-100	26.00 ± 1.23^a^	25.22 ± 1.66^a^	26.16 ± 0.11^a^
LC-200	25.83 ± 0.50^a^	22.73 ± 1.05^a^	25.38 ± 1.39^a^
LC-300	22.47 ± 2.08^a^	25.94 ± 1.04^a^	22.78 ± 2.61^b^

LC-100, LC-200, and LC-300 were supplemented L-carnitine at rates of 100, 200, and 300 mg L^−1^, respectively. Same superscripts in a column do not differ significantly ( P > 0.05).

**Table 8:** The effect of maternal L-carnitine supplementation on serum cortisol (ng L^−1^) concentration in rabbit neonates.

Groups	First week	Second week	Third week
Control	21.28 ± 1.95^a^	21.17 ± 1.39^a^	18.08 ± 0.81^a^
LC-100	22.66 ± 0.54^a^	19.93 ± 0.41^a^	17.01 ± 0.81^a^
LC-200	21.84 ± 0.28^a^	20.11 ± 0.68^a^	16.80 ± 0.32^a^
LC-300	23.00 ± 1.95^a^	20.40 ± 1.13^a^	18.34 ± 1.11^a^

LC-100, LC-200, and LC-300 were supplemented L-carnitine at rates of 100, 200, and 300 mg L^−1^, respectively. Same superscripts in a column do not differ significantly ( P > 0.05).

Serum cortisol concentration did not differ significantly among treatments during any sampling period (Table 8). Nevertheless, a slight numerical decline was observed in the second and third weeks compared with the first week across most groups.

Maternal L-carnitine supplementation significantly improved serum T3 concentration, particularly at the highest inclusion level (Table 9). The LC-300 group consistently recorded the highest T3 concentration during all 3 weeks, whereas the control and lower-dose groups showed lower values.

**Table 9:** The effect of maternal L-carnitine supplementation on serum triiodothyronine (T3) (ng mL^−1^) concentration in rabbit neonates.

Groups	First week	Second week	Third week
Control	0.53 ± 0.04^b^	0.74 ± 0.13^ab^	0.54 ± 0.11^b^
LC-100	0.50 ± 0.02^b^	0.52 ± 0.09^b^	0.54 ± 0.11_b_
LC-200	0.65 ± 0.09^ab^	0.72 ± 0.05^a^	0.52 ± 0.04_b_
LC-300	0.79 ± 0.07^a^	0.94 ± 0.13^a^	0.82 ± 0.03^a^

LC-100, LC-200, and LC-300 were supplemented L-carnitine at rates of 100, 200, and 300 mg L^−1^, respectively. Different superscripts in a column differ significantly ( P < 0.05).

**Table 10:** The effect of maternal L-carnitine supplementation on serum thyroxine, T4 (ng mL^−1^) concentration in rabbit neonates.

Groups	First week	Second week	Third week
Control	5.89 ± 0.99^a^	5.22 ± 0.89^ab^	3.65 ± 0.76^b^
LC-100	3.76 ± 0.11^b^	2.78 ± 0.33^b^	3.65 ± 0.76^b^
LC-200	4.94 ± 0.18^ab^	5.90 ± 0.67^a^	5.17 ± 0.94^a^
LC-300	6.00 ± 0.34^a^	5.78 ± 0.99^a^	5.54 ± 0.21^a^

LC-100, LC-200, and LC-300 were supplemented L-carnitine at rates of 100, 200, and 300 mg L^−1^, respectively. Different superscripts in a column differ significantly ( P > 0.05).

Serum thyroxine (T4) concentration was also influenced by treatment (Table 10). During the first week, the LC-300 and control groups showed higher T4 concentration than LC-100. In the second week, LC-200 and LC-300 maintained higher T4 levels compared with LC-100. By the third week, LC-200 and LC-300 remained superior to the control and LC-100 groups.

Serum malondialdehyde (MDA) concentration was not significantly altered by maternal L-carnitine supplementation during any sampling period (Table 11), indicating no marked treatment effect on lipid peroxidation status under the present conditions.

**Table 11:** The effect of maternal L-carnitine supplementation on serum malondialdehyde (MDA, nmol L^−1^) concentration in rabbit neonates.

Groups	First week	Second week	Third week
Control	6.31 ± 0.50^a^	6.45 ± 0.90^a^	6.35 ± 1.50^a^
LC-100	6.37 ± 0.00^a^	6.46 ± 1.00^a^	6.36 ± 1.00^a^
LC-200	6.32 ± 0.00^a^	6.34 ± 1.00^a^	6.34 ± 2.50^a^
LC-300	6.33 ± 0.50^a^	6.44 ± 1.50^a^	6.32 ± 4.00^a^

LC-100, LC-200, and LC-300 were supplemented L-carnitine at rates of 100, 200, and 300 mg L^−1^, respectively. Same superscripts in a column do not differ significantly ( P > 0.05).

## 4 Discussion

The present findings demonstrated that maternal LC supplementation improved growth performance and modified several metabolic and endocrine indices in rabbit neonates. These responses are biologically plausible because LC is a vitamin-like compound that facilitates the transport of long-chain fatty acids across the inner mitochondrial membrane for β -oxidation, thereby enhancing adenosine triphosphate (ATP) generation and metabolic efficiency (Ferrari et al., 2004; Sawicka et al., 2020). In addition, LC can spare lysine and methionine for protein synthesis, improve glucose utilisation through pyruvate dehydrogenase activation, and reduce oxidative stress by stabilising mitochondrial function. These combined effects may explain the beneficial responses observed in offspring born to supplemented does (Hassan et al., 2024). Because maternal L-carnitine readily influences fetal and neonatal energy metabolism, improved mitochondrial fatty acid oxidation during gestation and lactation may enhance metabolic programming of offspring. This programming enables neonatal tissues to utilise both fatty acids and glucose more efficiently during the rapid transition from intrauterine to postnatal life, thereby supporting growth and metabolic adaptation. Furthermore, maternal L-carnitine is transferred to the developing fetus through the placenta and subsequently to neonatal rabbits through milk during lactation. Because fetal and neonatal endogenous synthesis of L-carnitine is limited, maternal supply represents the principal source of this metabolite during early life. Consequently, maternal supplementation may increase tissue carnitine availability in offspring, thereby enhancing mitochondrial fatty acid oxidation, energy production, and metabolic programming during critical stages of development (Harmeyer, 2002; Servillo, et al., 2018).

The higher individual body weight and average daily gain in neonates from LC-supplemented dams suggest improved prenatal and postnatal nutrient transfer, as well as enhanced milk quality or milk yield. Carnitine is naturally abundant in milk and is particularly important during early life because neonates have immature endogenous synthesis and rely partly on maternal supply (Servillo et al., 2018). In addition to milk, placental transport of L-carnitine during gestation contributes to fetal tissue accumulation before birth, while continued transfer through colostrum and milk helps maintain neonatal carnitine stores during the suckling period. This continuous maternal supply supports efficient utilisation of milk lipids as an energy source and facilitates the rapid metabolic adaptation required immediately after birth (Harmeyer, 2002; Servillo et al., 2018). Increased maternal LC availability may therefore improve neonatal fatty acid oxidation, allowing more efficient use of milk lipids for growth rather than fat storage (Harmeyer, 2002). Enhanced mitochondrial energy production also supports intestinal development and nutrient absorption, which contributes to faster growth. Similar growth-promoting effects of LC have been reported in growing rabbits, where supplementation improved final body weight, body weight gain, feed conversion ratio, and production index, particularly at moderate doses (Hassan et al., 2024). Importantly, these findings are consistent with emerging evidence that maternal nutritional interventions can programme neonatal growth and health through coordinated metabolic, endocrine, and intestinal pathways (Xie et al., 2023).

The superior response at intermediate or higher doses in the current study may indicate a dose-dependent improvement in maternal nutrient partitioning. However, extremely high LC levels do not always yield additional benefits as some studies reported optimal performance at moderate inclusion rates rather than maximal doses. This suggests that LC responses depend on physiological stage, diet composition, and environmental stress.

The increase in serum glucose, especially in neonates from the LC-300 group, indicates enhanced carbohydrate metabolism and improved energy homeostasis. LC indirectly regulates glucose oxidation by buffering acetyl-CoA / CoA ratios in mitochondria and activating pyruvate dehydrogenase, thereby facilitating the entry of glycolytic products into the tricarboxylic acid cycle. Improved maternal energy status may also enhance milk lactose synthesis and glucose supply to offspring. Elevated glucose in early life may reflect better nutrient availability rather than metabolic dysfunction, particularly when accompanied by improved growth. Similar evidence in rabbits and poultry has shown that LC can improve metabolic efficiency under stress conditions by enhancing both lipid and carbohydrate utilisation (Hassan et al., 2024). In agreement, LC supplementation in growing rabbits has been shown to increase blood glucose and total protein concentrations, suggesting improved nutrient metabolism and systemic energy status, particularly under challenging environmental conditions (Harmeyer, 2002; Servillo et al., 2018; Ayyat et al., 2021; Cioffi et al., 2022). These findings are further supported by reports showing that maternal dietary interventions can enhance intestinal glucose transport and metabolic efficiency in neonates, contributing to improved energy utilisation (Servillo et al., 2018). Mechanistically, enhanced mitochondrial β -oxidation reduces reliance on anaerobic glycolysis while simultaneously increasing the efficiency of glucose oxidation through the tricarboxylic acid cycle. Consequently, circulating glucose is more likely to reflect improved nutrient availability and metabolic flexibility rather than impaired glucose regulation.

The reduction or stabilisation of serum cholesterol in LC-supplemented groups indicates improved lipid metabolism. LC increases β -oxidation of fatty acids, reducing hepatic lipid accumulation and decreasing substrate availability for cholesterol synthesis. Another proposed mechanism is the binding of acetyl groups into acyl-carnitine forms, limiting acetyl-CoA availability for cholesterogenesis. In the attached rabbit study, LC supplementation significantly lowered total cholesterol and LDL while improving HDL, confirming its hypolipidemic effect (Sawicka et al., 2020). Similarly, maternal supplementation strategies have been shown to reduce LDL-C and improve lipid profiles in both dams and neonates, indicating improved systemic lipid metabolism (Servillo et al., 2018).

The absence of significant differences in triglycerides in the present study is also consistent with previous rabbit data where triglycerides sometimes remained unchanged despite improvements in cholesterol fractions. This may occur because triglyceride concentration is strongly influenced by recent nutrient intake and age-related metabolic shifts, whereas cholesterol more clearly reflects sustained hepatic lipid regulation.

AST and ALT are widely used indicators of hepatic integrity. The reduction in AST and ALT, particularly in the LC-300 group during later weeks, suggests improved liver health and reduced hepatocellular stress. LC enhances mitochondrial fatty acid oxidation, preventing lipid infiltration of hepatocytes and reducing reactive oxygen species formation. By maintaining membrane stability and cellular energy balance, LC may lower the leakage of intracellular enzymes into circulation. Comparable observations in rabbits receiving LC or metabolic enhancers have shown improved liver function profiles and lower serum enzyme activities, supporting the hepatoprotective role of LC (Sawicka et al., 2020). This improvement in hepatic function is consistent with enhanced antioxidant status reported in maternal supplementation studies, where increased activities of enzymes such as glutathione peroxidase were associated with reduced oxidative damage (Servillo et al., 2018). Improved hepatic mitochondrial function may also increase ATP availability for cellular repair processes and maintenance of hepatocyte membrane integrity, thereby minimising enzyme leakage into the bloodstream. These findings suggest that maternal L-carnitine not only supports hepatic energy metabolism but also contributes to the preservation of normal liver function during early neonatal development. Because neonatal rabbits undergo rapid metabolic transition after birth, liver support through maternal supplementation may be particularly valuable during the first weeks of life.

The lack of significant treatment effect on cortisol indicates that maternal LC did not markedly alter basal hypothalamic–pituitary–adrenal axis activity. Nevertheless, the numerical tendency toward lower cortisol in some supplemented groups may reflect reduced physiological stress and better adaptation. LC has been associated with improved energy balance and antioxidant defence, both of which can blunt stress responses. However, under non-stressful rearing conditions, baseline cortisol may remain within normal limits, masking treatment differences. Similar observations have been reported where maternal dietary interventions improved immune and inflammatory status without significantly altering baseline stress hormone levels (Servillo et al., 2018). Therefore, the absence of significant cortisol changes should be interpreted as the maintenance of normal endocrine homeostasis rather than a lack of biological response. It is possible that the beneficial effects of L-carnitine become more evident only under environmental, nutritional, or physiological stress conditions that activate the hypothalamic–pituitary–adrenal axis.

The marked increase in T3 and improved T4 status in neonates from LC-supplemented dams indicates enhanced thyroid activity and metabolic rate. Thyroid hormones stimulate oxygen consumption, thermogenesis, protein synthesis, and growth; therefore, their elevation aligns with the higher body weight and daily gain observed. LC may influence thyroid hormones indirectly by improving mitochondrial responsiveness and energy demand, which can stimulate peripheral conversion of T4 to the more active T3 (Surai, 2015). Improved hepatic function may also support deiodinase enzyme activity. In growing rabbits treated with metabolic agents including LC, improvements in thyroid hormone profile have also been reported, supporting the link between LC supplementation and endocrine stimulation (Hassan et al., 2024). The consistently highest T3 values in the LC-300 group suggest that higher maternal LC intake may intensify metabolic signalling during neonatal growth. More broadly, maternal nutritional strategies have been shown to modulate neonatal endocrine profiles, including growth-related hormones, highlighting the role of maternal diet in early-life metabolic programming (Servillo et al., 2018). The elevated T3 concentration is particularly important because T3 is the biologically active thyroid hormone responsible for stimulating mitochondrial biogenesis, oxidative phosphorylation, and cellular oxygen consumption. Consequently, increased T3 may have acted synergistically with L-carnitine-mediated fatty acid transport to maximise mitochondrial ATP production, thereby supporting rapid tissue growth and improved feed energy utilisation in neonatal rabbits. These endocrine responses are likely to be facilitated by adequate maternal transfer of L-carnitine during gestation and lactation, ensuring sufficient substrate availability for mitochondrial energy metabolism in rapidly developing neonatal tissues.

MDA is a marker of lipid peroxidation and oxidative stress. The absence of significant changes among groups indicates that oxidative challenge may have been low under the present management conditions. When baseline oxidative stress is minimal, LC supplementation may maintain rather than dramatically reduce MDA. Nonetheless, LC is known to possess antioxidant actions through improved mitochondrial efficiency, reduced free radical generation, and enhanced endogenous antioxidant systems (Vimani and Cirull, 2022). In another rabbit maternal nutrition study, nutritional supplementation significantly reduced neonatal MDA and improved catalase and glutathione peroxidase activities, demonstrating that antioxidant responses can be detected when oxidative load is greater. In contrast, studies under higher oxidative challenge conditions have reported significant reductions in MDA along with increases in antioxidant enzymes such as catalase and glutathione peroxidase following maternal supplementation (Mpwaga et al., 2026). Thus, the unchanged MDA in the present study should not be interpreted as a lack of LC efficacy but rather as evidence that lipid peroxidation was already controlled. Furthermore, maintenance of low MDA concentrations, together with improved growth performance and liver function, suggests that maternal L-carnitine enhanced mitochondrial efficiency without inducing oxidative damage. This finding supports the concept that improved mitochondrial fatty acid oxidation can increase energy production while maintaining redox homeostasis, thereby preserving cellular integrity during the neonatal period. However, because only MDA was determined in the present study, the antioxidant status of the neonatal rabbits cannot be comprehensively characterised. Assessment of enzymatic antioxidant biomarkers, including glutathione peroxidase (GPx), superoxide dismutase (SOD), and catalase (CAT), would have provided a more complete evaluation of the endogenous antioxidant defence system. Therefore, the present findings should be interpreted as indications of the fact that maternal L-carnitine supplementation did not increase lipid peroxidation under the prevailing experimental conditions rather than as confirmation of an overall enhancement of antioxidant capacity. Future studies incorporating both enzymatic and non-enzymatic oxidative stress biomarkers are warranted to further elucidate the antioxidant mechanisms associated with maternal L-carnitine supplementation.

## 5 Conclusion

Maternal L-carnitine supplementation at 300 mg L^−1^ improved neonatal growth performance and was associated with enhanced glucose metabolism and thyroid hormone concentrations. Serum AST and ALT activities remained within the normal physiological range, indicating that maternal L-carnitine supplementation did not adversely affect hepatic integrity. Furthermore, lipid profile, cortisol, and MDA concentrations were not altered, suggesting that supplementation was well tolerated under the conditions of the present study. Overall, these findings support the potential use of maternal L-carnitine supplementation as a nutritional strategy to promote neonatal growth and metabolic development in rabbits. From a practical perspective, supplementation at 300 mg L^−1^ may represent a promising nutritional intervention for commercial rabbit production by improving offspring performance; however, its adoption should be supported by cost–benefit analyses to determine its economic feasibility under commercial farming conditions. Additionally, further studies including additional markers of antioxidant status and liver function are warranted to confirm the underlying mechanisms.

## Supplement

10.5194/aab-69-585-2026-supplement The supplement related to this article is available online at https://doi.org/10.5194/aab-69-585-2026-supplement.

## Data Availability

The data are available from the corresponding author on request.
