# Supplementary material for: Carry-over influence of maternal L-carnitine during gestation and lactation on metabolic response of New Zealand neonatal rabbits
Source: Arch Anim Breed. 2026 Oct 19;69(4):585–94. doi: 10.5194/aab-69-585-2026 (PMC13681338; doi:10.5194/aab-69-585-2026)
Supplement: Supplementary file 1 [file aab-69-585-2026-supplement.pdf]

Supplement of Arch. Anim. Breed., 69, 585–594, 2026
https://doi.org/10.5194/aab-69-585-2026-supplement
© Author(s) 2026. CC BY 4.0 License.

Open Access
Archives Animal Breeding

Supplement of

Carry-over influence of maternal L-carnitine during gestation and lactation on metabolic response of New Zealand neonatal rabbits

Shahzada Khurram Adrian Shah et al.

Correspondence to: Shabana Naz (drshabananaz@gcuf.edu.pk) and Rifat Ullah Khan (rukhan@aup.edu.pk)

The copyright of individual parts of the supplement might differ from the article licence.

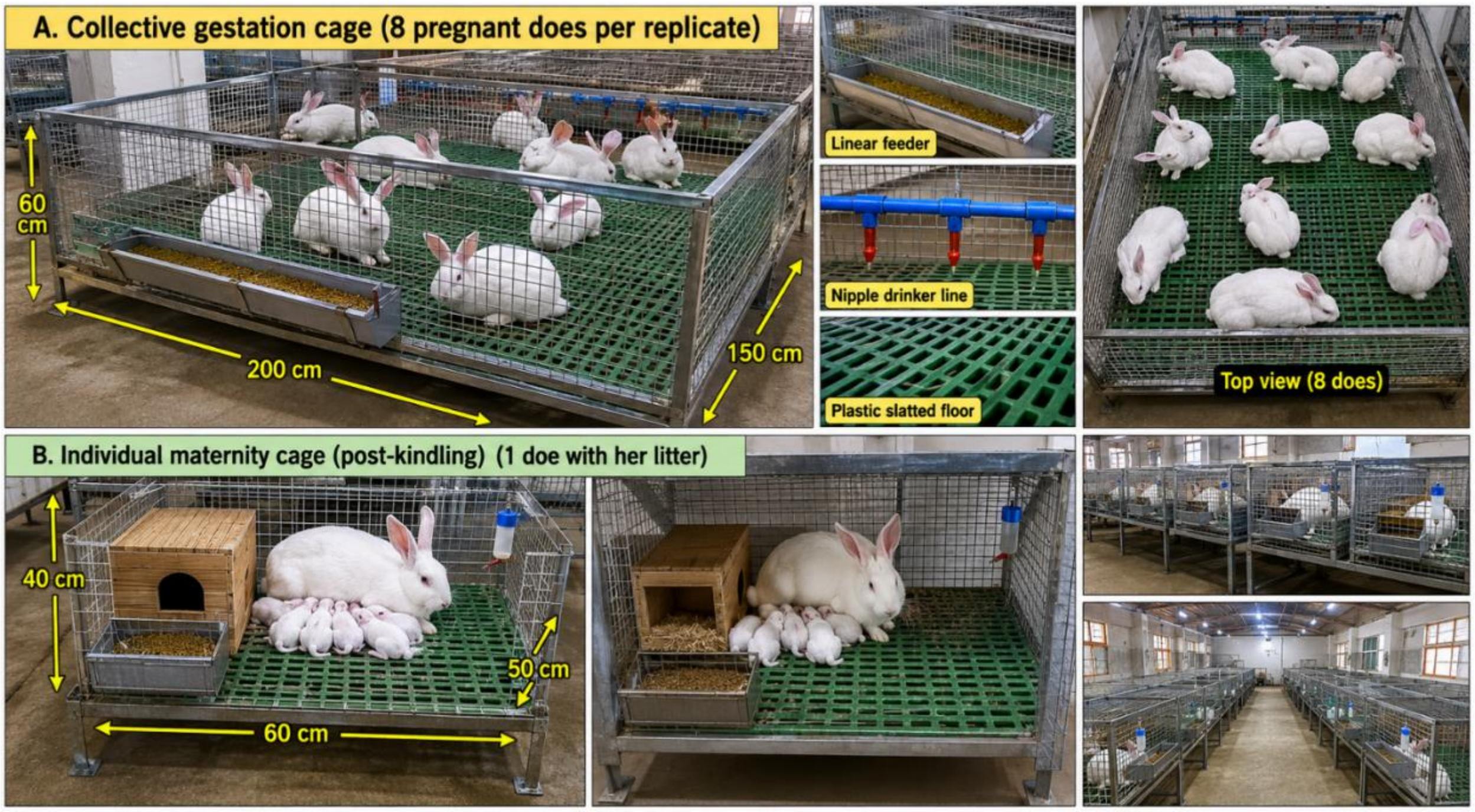

Fig S1: Housing facilities used for maternal rabbits during gestation and lactation. (A) Collective gestation cage accommodating eight pregnant does per replicate, showing the linear feeder, nipple drinker line, plastic slatted floor, and top view of the cage. (B) Individual maternity cage used after kindling, with one doe and her litter, showing the nest box, feeder, and nipple drinker.
